# Texture design for microwave dielectric (Ca_0.7_Nd_0.3_)_0.87_TiO_3_ ceramics through reactive-templated grain growth

**DOI:** 10.1088/1468-6996/16/3/035008

**Published:** 2015-06-04

**Authors:** Toshihiko Tani, Tsuguto Takeuchi

**Affiliations:** 1 Toyota Central Research and Development Laboratories, Inc., Nagakute, Aichi, 480-1192, Japan; 2 Toyota Technological Institute, Nagoya, 468-8511, Japan

**Keywords:** microwave dielectric, textured ceramic, reactive template, grain boundary orientation

## Abstract

Plate-like Ca_3_Ti_2_O_7_ (CT) and Nd_2_Ti_2_O_7_ (NT) particles were synthesized in molten salts and used as reactive templates for the preparation of highly textured (Ca_0.7_Nd_0.3_)_0.87_TiO_3_ bulk ceramics (CNT) with preferred pseudocubic 〈100〉 and 〈110〉 orientations, respectively. During flux growth CT and NT particles developed facets parallel to the pseudocubic {100} and {110} planes, respectively, in a perovskite unit cell, since those planes correspond to the interlayers of the layered perovskite-type crystal structures. Complementary reactants for the CNT stoichiometry were wet-mixed with the reactive templates and the slurries were tape-cast. Then stacked tapes were heat-treated for dense single-phase CNT ceramics with a distorted and A-site deficient regular perovskite-type structure. The CNT ceramics prepared with CT and NT reactive templates exhibited strong pseudocubic 100- and 110-family x-ray diffraction peaks, respectively, with other peaks drastically suppressed when non-perovskite sources were used as complementary reactants. The textured ceramics possess unique microstructures; as either parallel or obliquely stacked block structures with a pseudocubic {100} plane faceted. The pseudocubic {100}-and {110}-textured CNT ceramics exhibited ∼10 and ∼20% higher products of the dielectric quality factor and frequency, *Q* · *f*, respectively, than conventional ceramic sintered at the same temperature. When *Q* · *f* is compared based on the same grain size, the {100}-textured CNT exhibited 27% higher values than non-textured while relative permittivity and temperature coefficient of resonant frequency were of similar values. Simple geometrical relationships between electric field and penetrated pseudocubic {*hk*0}-type grain boundaries must lead to the reduced scattering and dielectric loss.

## 1. Introduction

Texture engineering is an approach to enhance mechanical and/or functional performances of polycrystals for materials with anisotropic properties. Processing methods for textured bulk ceramics are largely restricted to hot-working [1], colloidal processing in magnetic field [2, 3] and the use of oriented anisometric particles, such as templated grain growth (TGG) [4, 5], because of limited applicability of melting/recrystallization and plastic deformation techniques for ceramics unlike for metals and plastics. Texturing process is especially limited for materials in pseudo-cubic crystal systems such as a regular perovskite-type structure. Piezoelectric properties have been reported to be enhanced for regular perovskite-type materials such as Bi_0.5_Na_0.5_TiO_3_- [6], PbTiO_3_- [7] and K_0.5_Na_0.5_NbO_3_-based systems [8] with preferred 〈100〉_pc_ orientation where ‘pc’ stands for a direction or a plane expressed in pseudocubic notation. These textured ceramics with a regular perovskite-type were processed by exploiting topochemical conversion either for the preparation of anisometric regular perovskite-type particles for TGG from isomorphic precursors [8, 9] or for *in situ* formation of regular perovskite-type grains from aligned anisometric precursor particles with complementary reactants [10]. The latter processing is named as reactive-templated grain growth (RTGG) and Aurivillius-type and Ruddlesden–Popper-type materials are proposed as reactive templates for {100}_pc_ texture [6, 11] whereas limited number of approaches have been known for textured bulk ceramics with other preferred orientations [12]. We need a texture engineering method to ‘tailor’ a ceramic with a particular preferred orientation direction in which the best performance can be extracted from a material. Texture control was reported on perovskite-type lead zirconate with preferred 〈100〉- and 〈111〉-orientations, in sol–gel derived thin films on Pt/Ti/SiO_2_/Si substrates, by chemical modifications of solutions and substrate surfaces [13]. Drastic changes in the polarization-electric field hysteresis behaviors were reported according to the preferred-orientation direction. In bulk ceramics, however, few reports have been known for the design and fabrication of pseudocubic materials with different preferred orientation directions, even for thick films [14].

Texture engineering has been applied to microwave dielectric ceramics with anisotropic crystal structures for the improvement of permittivity and temperature coefficient [15]. For pseudocubic regular perovskite-type materials, Saito *et al* reported the fabrication of a {100}_pc_-textured CaTiO_3_ ceramic by TGG method and its 55% larger figure of merit, *Q* · *f*, than a randomly oriented ceramic [16]. Here *f* is frequency and *Q* is the dielectric quality factor, taken as the inverse of the dielectric loss tangent, 1/tan*δ*. They attributed the improved *Q* · *f* value to a reduced scattering loss at grain boundaries in the {100}_pc_-textured ceramic. It is not clear, however, whether the improved performance is especially due to the {100}_pc_ or any types of texture. It must be, thus, an important subject to fabricate ceramics with the same composition and different preferred orientation directions for the investigation of the dielectric properties. We have made a preliminary report on the fabrication of textured (Ca_0.7_Nd_0.3_)_0.87_TiO_3_ ceramics and their microwave dielectric properties [17]. In this paper we describe the chemical design strategy for the preparation of ceramics with tailored textures through *in situ* topochemical conversion reactions on two different types of reactive templates. Preparation, characterization and property measurements were conducted for {100}_pc_- and {110}_pc_-textured ceramics with the same target composition and the relationship between microstructures and microwave dielectric properties are discussed.

## 2. Reaction designs for textured polycrystals

A-site deficient, distorted perovskite-type (Ca_0.7_Nd_0.3_)_0.87_TiO_3_ (or Ca_0.61_Nd_0.26_TiO_3_, abbreviated as CNT) was selected as a target material for texture control. This composition of ceramics has been known to have a combination of high relative permittivity, *∊*_r_ (∼100), and fairly good *Q* · *f* value (∼17 000 GHz) [18, 19].

**Figure 1.**
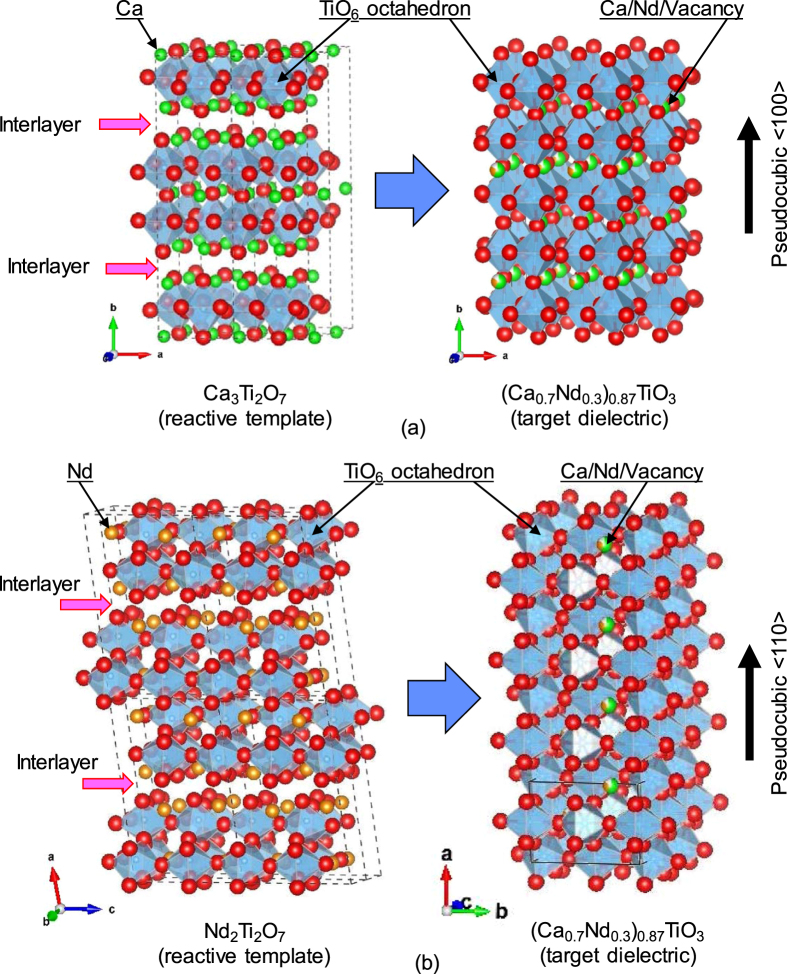
Crystal structures of (a) (100)-type layered perovskite Ca_3_Ti_2_O_7_, and (b) (110)-type layered perovskite Nd_2_Ti_2_O_7_, schematically shown with crystal structures of distorted regular perovskite (Ca_0.7_Nd_0.3_)_0.87_TiO_3_ with their TiO_6_ octahedrons approximately aligned in the same directions as the layered perovskite reactive templates.

Ruddlesden–Popper-type Ca_3_Ti_2_O_7_ (CT) is an isomorph of Sr_3_Ti_2_O_7_ which was synthesized in a plate-like shape by a molten salt synthesis (MSS) and used as a template for {100}_pc_-textured regular perovskite-type ceramics [11, 20]. Ruddlesden–Popper-type layered perovskite has an interlayer parallel to orthorhombic {010}, i.e., {100}_pc_ for a perovskite unit cell, where the periodic bond chain (PBC),-Ti–O–Ti–O-,is terminated with a corner of TiO_6_ octahedron. The equilibrium shape of a crystal in this family would be plate-like with its interlayer as a developed plane [9]. Thus the plate-like CT particles, if aligned with the developed plane parallel to one another, could be converted topochemically into the regular perovskite with its {100}_pc_ parallel to the original interlayer of the reactive template, schematically described in figure 1(a) [21].

On the other hand, Nd_2_Ti_2_O_7_ (NT) has a strontium pyroniobate (Sr_2_Nb_2_O_7_)-type structure which has an interlayer parallel to {110}_pc_ for a perovskite unit cell, where the PBC is terminated with a edge of TiO_6_ octahedron. Brahmaroutu *et al* reported that Sr_2_Nb_2_O_7_ particles prepared by MSS exhibited elongated plate-like morphology [22]. Plate-like Sr_2_Nb_2_O_7_ particles were reported to be used as reactive templates for regular perovskite-type ceramics with a preferred {110}_pc_ [23]. If NT particles also show plate-like morphology similarly to its isomorphic compound, they are also expected to be converted topochemically into a regular perovskite with its {110}_pc_ parallel to the original interlayer of the reactive template, schematically described in figure 1(b) [21].

As complementary reactants for the formation of CNT, Nd-and Ti-source materials must be mixed with CT platelets for the {100}_pc_ texture, whereas Ca-and Ti-source materials are needed with NT platelets for the {110}_pc_ texture. Three types of reaction schemes were designed with the use of reactive templates (denoted as ‘|_rt_’):

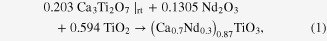


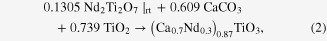


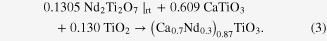


The reaction schemes (1) and (2) were designed so that the complementary reactants for A-site and B-site are supplied separately. The reaction scheme (3) was designed, to the contrary, with the use of maximum amount of complex oxide and minimum amount of simple oxide/carbonate, for the preparation of dense ceramics with minimized formation of Kirkendall voids. In the reaction (1), 40.6% of titanium is supplied by the reactive template CT, whereas 26.1% of titanium is given by the reactive template NT for the reaction (2) and (3).

## 3. Experimental procedures

### 3.1. Preparation of reactive template particles

Two types of plate-like reactive template particles were prepared by MSS in a mixed KCl + NaCl flux. CaCO_3_ (Kojundo Chemical Lab., 99% up) and TiO_2_ (Ishihara Sangyo, Tipaque A-100, anatase, 100 nm, 99% up) were mixed with a Ca/Ti ratio of 3/2 in ethanol by ball milling for Ruddlesden–Popper-type CT. Dried powders were then mixed with the equal weight of 0.5 NaCl + 0.5 KCl as a flux and heat-treated in a platinum crucible at 1673 K for 8 h. Nd_2_O_3_ (Kojundo Chemical Lab., 99.9%) and TiO_2_ were mixed with an Nd/Ti ratio of 1/1 in ethanol by ball milling for strontium-pyroniobate-type NT. Dried powders were similarly mixed with the 0.5 NaCl + 0.5 KCl flux and heat-treated in a platinum crucible at 1573 K for 8 h. Products were immersed in deionized water at 353 K and the flux was removed by repeated filtration until chlorine could not be detected by AgNO_3_ solution. Equiaxed CT and NT particles were also prepared as reference specimens by solid-state reactions (SSRs) of CaCO_3_ and TiO_2_, and Nd_2_O_3_ and TiO_2_, respectively, without flux.

### 3.2. Preparation and characterization of textured CNT polycrystals

Table 1 lists the source materials and compositions used for the preparation of CNT ceramics. Conventionally processed CNT ceramics (CP-CNT) were prepared by sintering of CNT powder compacts synthesized by an SSR. The SSR–CNT powder was prepared by heating mixed powder compacts of CaCO_3_, Nd_2_O_3_ and TiO_2_ with a stoichiometric ratio (Ca:Nd:Ti = 0.609:0.261:1) at 1473 K for 8 h and pulverized by ball-milling. Die-pressed (at 25 MPa) and cold-isostatic-pressed (at 300 MPa) SSR–CNT powder compacts were sintered at 1673–1823 K for 10 h in air for the preparation of CP-CNT with different grain sizes.

**Table 1.** Molar ratios of materials used for four batches of (Ca_0.7_Nd_0.3_)_0.87_TiO_3_ ceramics.

Batch	MSS–CT^a^	MSS–NT	SSR–CNT	SSR–CaTiO_3_	CaCO_3_	Nd_2_O_3_	TiO_2_
CP-CNT	—	—	1	—	—	—	—
CT–R–CNT	0.2256	—	—	—	—	0.1305	0.5489
NT–R–CNT	—	0.1305	—	—	0.609	—	0.739
NT–F–CNT	—	0.1305	—	0.609	—	—	0.130

a Chemical formula of MSS–CT (a mixture of Ca_3_Ti_2_O_7_ and Ca_4_Ti_3_O_10_ platelets) was assumed as Ca_2.7_Ti_2_O_6.7_ for the calculation, based on ICP analysis (Ca/Ti = 1.35).

For textured CNT, three batches of slurries were prepared by mixing molten-salt-synthesized plate-like particles with complementary reactants as well as polyvinyl butyral (PVB) as a binder and di-n-butyl phthalate (DBP) as a plasticizer dissolved in a mixed toluene–ethanol solvent. Plate-like calcium titanate, MSS–CT, identified as a mixture of Ca_3_Ti_2_O_7_ and Ca_4_Ti_3_O_10_ particles, was used as a reactive template while Nd_2_O_3_ and TiO_2_ were used as complementary reactants for CT–R–CNT. Here ‘R’ stands for a specimen prepared with ‘raw material-type’ complementary reactant(s). A Ca/Ti ratio of 1.35, determined by inductively coupled plasma atomic emission spectrometry (ICP-AES) analysis, was used for the MSS–CT for the calculation of starting powder ratio. Plate-like Nd_2_Ti_2_O_7_ particles, MSS–NT, were used as a reactive template for NT–R–CNT and NT–F–CNT where ‘F’ stands for a specimen prepared with ‘filler-type’ complementary reactant(s). As complementary reactants, CaCO_3_ and TiO_2_ were used for NT–R–CNT whereas pre-synthesized equiaxed CaTiO_3_ powder by a SSR (heated at 1473 K for 8 h in air and pulverized by ball-milling) was used with TiO_2_ for NT–F–CNT. The slurries were tape-cast by a doctor blade technique into ∼0.1 mm thick tapes and the tapes were stacked at 353 K at 10 MPa into 1 mm thick green bodies. The alignment of the developed planes of the reactive template particles was confirmed by x-ray diffraction (XRD) for the tape-casting surfaces of the green bodies. The green bodies were dewaxed at 873 K for 2 h and sintered at 1773 K for 10 h in air.

The densities of sintered specimens were evaluated by the Archimedes method and compared with calculated values from lattice parameters which were determined by XRD. Phase development and texture were characterized by XRD for the tape-cast plane after the removal of as-fired surfaces. Texture was evaluated in terms of the Lotgering’s factor, *F* = (*p* − *p*_0_)/(1 − *p*_0_), where *p* = *ΣI*_(*h*00)pc_/*ΣI*_(*hkl*)pc_ for {100}_pc_-textured ceramics and *p* = *ΣI*_(*hh*0)pc_/*ΣI*_(*hkl*)pc_ for {110}_pc_-textured ceramics, and *p*_0_ = *p* (*F* = 0) for randomly oriented ceramics [24]. The perpendicular sections of the sintered specimens to the original tape surfaces were mirror-polished and thermally etched at 1473–1523 K for 30 min in air for the observation of microstructures in terms of grain sizes and morphologies by scanning electron microscopy (SEM). The average grain sizes of the ceramics were estimated by the intercept method.

### 3.3. Measurements of dielectric properties

**Figure 2.**
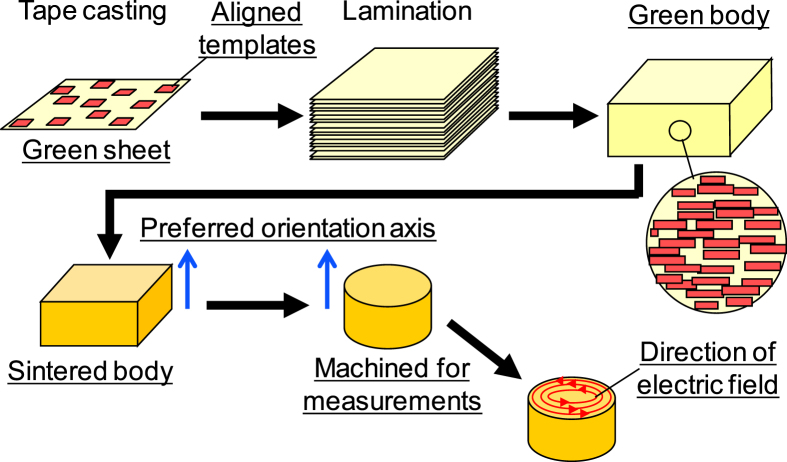
Schematic relationships between tape-cast green sheets and sintered ceramic before and after machining into a cylinder for microwave dielectric measurements.

For microwave dielectric measurements, secondary lamination of 1 mm thick green bodies and subsequent sintering produced 10 mm thick specimens for highly textured CNT ceramics as well as randomly oriented CP-CNT ceramics. Cylindrical specimens 8.2 mm in diameter and 4.1 mm in thickness were machined out from the sintered bodies with their cylinder axes perpendicular to the original tape surfaces, as schematically shown in figure 2. Hakki–Coleman and Kobayashi methods [25, 26] were used to determine the microwave dielectric quality factor *Q* and relative permittivity *∊*_r_ in the TE_011_ mode at frequencies ∼5 GHz with a network analyzer (HP, 8757 A) at 298 K. Temperature coefficient of resonant frequency *τ*_f_ was measured between 298 and 353 K. It should be noted that the electric field is in the circular direction which is parallel to the original tape surfaces and perpendicular to the orientation axis of textured ceramics.

## 4. Results and discussion

### 4.1. Morphologies and phases of flux-grown particles

**Figure 3.**
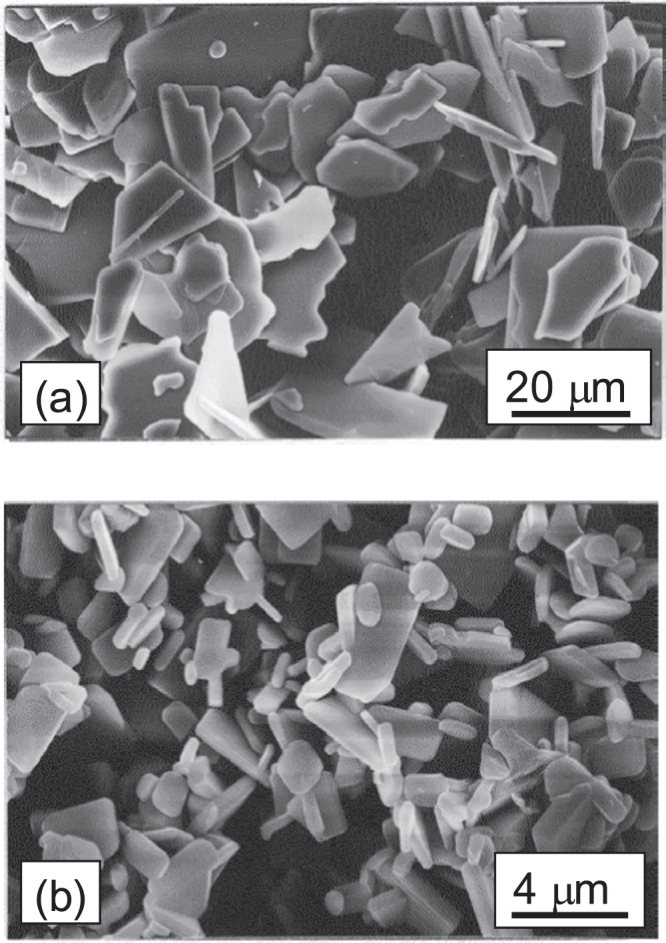
SEM images of flux-grown particles. (a) MSS–CT particles prepared at 1673 K for 8 h. (b) MSS–NT particles prepared 1573 K for 8 h.

Figure 3 shows SEM images of flux-grown particles. MSS–CT particles show plate-like morphology with disordered shapes in their developed plane with sizes of 10–20 *μ*m. MSS–NT particles show business card-like morphology with their developed plane sizes of 1–5 *μ*m. The aspect ratios of the plate-like particles were approximately ∼20 and ∼10 for MSS–CT and MSS–NT, respectively.

XRD analysis showed that MSS–NT was single-phase Nd_2_Ti_2_O_7_, whereas MSS–CT was composed of Ca_3_Ti_2_O_7_ and Ca_4_Ti_3_O_10_, both Ruddlesden–Popper-type-structured compounds with two and three-layers of perovskite units, respectively. Synthesis conditions for single-phase Ca_3_Ti_2_O_7_ were not found in the preliminary MSS experiment, for the MSS–CT products synthesized at 1473 and 1573 K for 8 h were single-phase CaTiO_3_ and mixed phases of CaTiO_3_ and Ca_4_Ti_3_O_10_, respectively, whereas a SSR from the same mixed powders of Ca/Ti = 3/2 without flux produced phase-pure Ca_3_Ti_2_O_7_. The Ca/Ti atomic ratio for the MSS–CT particles was determined as 1.35 by ICP-AES and this value was used for the reaction design for RTGG processing as mentioned previously.

### 4.2. Textures and microstructures of ceramics

**Figure 4.**
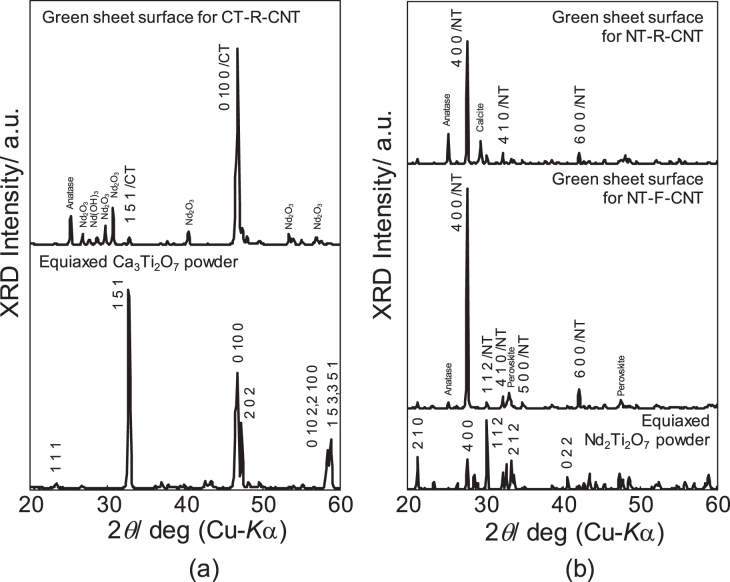
XRD patterns for (a) the green sheet surface of CT–R–CNT as compared with equiaxed Ca_3_Ti_2_O_7_ particles prepared by a solid-state reaction, and (b) the green sheets surfaces of NT–R–CNT and NT–F–CNT as compared with equiaxed Nd_2_Ti_2_O_7_ particles prepared by a solid-state reaction.

Figure 4 shows XRD patterns for the tape casting surfaces of the CT–R–CNT and NT–R–CNT green sheets, as compared with the powder diffraction patterns of equiaxed CT and NT prepared by SSRs. It is apparent that the {010} plane of CT and the {100} plane of NT were aligned parallel to the sheet surfaces by a shear stress during tape-casting. The results indicate that they are developed planes of the plate-like template particles, as expected.

Table 2 lists the relative density, average grain size and Lotgering’s degree of orientation of the sintered specimens. All the sintered ceramics exhibited similar relative densities ranging between 96.8 and 97.8%. The lowest value (96.8%) was measured for NT–R–CNT which used a large amount of CaCO_3_ as a Ca source. Instead, NT–F–CNT with the use of pre-synthesized perovskite CaTiO_3_ as a Ca source exhibited improved density (97.6%) as designed.

**Table 2.** Relative density, grain size and Lotgering’s orientation degree *F* of (Ca_0.7_Nd_0.3_)_0.87_TiO_3_ ceramics.

Batch	Sintering temperature (K)	Relative density (%)	Average grain size (*μ*m)	Lotgering’s *F* for (*hkl*)_pc_^a^
CP-CNT	1673	97.6	47	—
CP-CNT	1773	97.8	72	—
CP-CNT	1823	97.2	93	—
CT–R–CNT	1773	97.2	40	0.998 for {100}_pc_
NT–R–CNT	1773	96.8	110	0.983 for {110}_pc_
NT–F–CNT	1773	97.6	73	0.184 for {110}_pc_

a Lotgering’s orientation degree was estimated from the XRD patterns for the polished surfaces parallel to the original tape surfaces.

**Figure 5.**
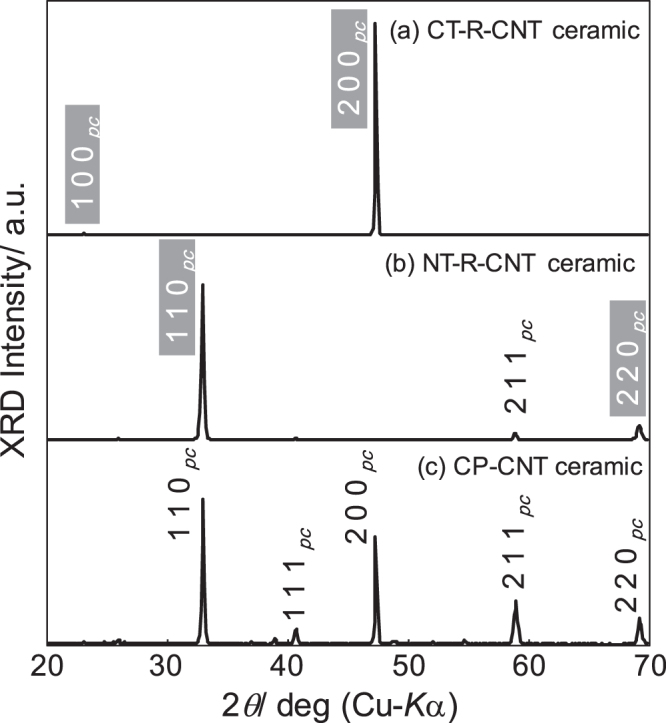
XRD patterns for the RTGG-processed ceramics of (a) CT–R–CNT and (b) NT–R–CNT on the polished surfaces parallel to the original tape surfaces as well as XRD pattern for (c) the conventionally processed CP-CNT ceramic. All the ceramics were sintered at 1773 K.

Figure 5 shows XRD patterns for the sintered ceramics of CT–R–CNT and NT–R–CNT on the polished surfaces parallel to the original tape surfaces as well as an XRD pattern for the conventionally processed CP-CNT ceramic. It should be noted that (100)_pc_ and (110)_pc_ corresponds to (101) and (020), and (200) and (121), respectively, in orthorhombic notation. All the sintered specimens are single-phase distorted and A-site deficient regular-perovskite CNT. It is remarkable that CT–R–CNT exhibits strong {100}_pc_-family XRD peaks whereas NT–R–CNT is highly {110}_pc_-textured, with the other diffraction peaks were drastically suppressed.

**Figure 6.**
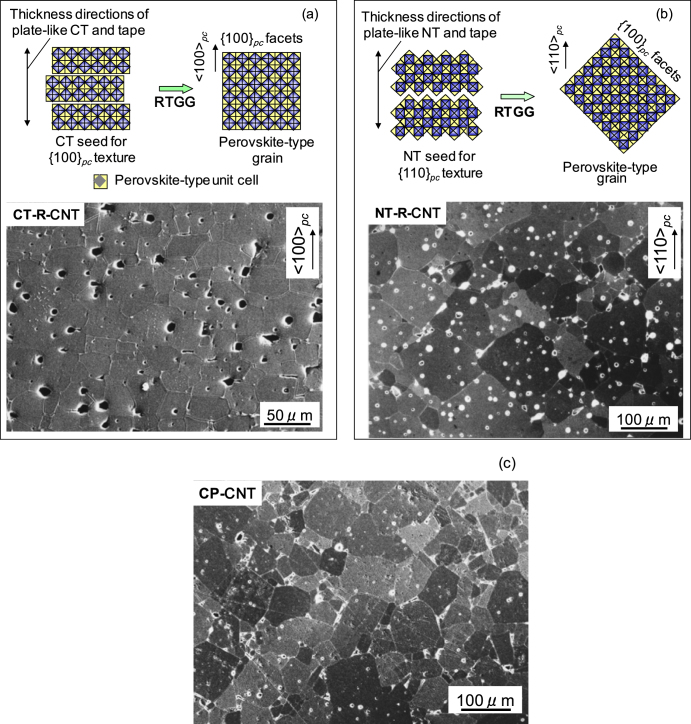
SEM images of (a) {100}_pc_-textured CT–R–CNT and (b) {110}_pc_-textured NT–R–CNT ceramics for the polished and thermally etched sections that were perpendicular to the original tape surfaces as well as (c) conventionally processed CP-CNT ceramic, sintered at the same temperature. Schematic diagrams for the formation of aligned grains with {100}_pc_ facets are also displayed.

The textured ceramics display unique microstructures on the sections perpendicular to the original tape surfaces when compared with a microstructure of conventionally processed CP-CNT with random orientation (figure 6(c)). The {100}_pc_-textured CNT ceramic shows a microstructure with parallel aligned block-shaped grains, or a brick-wall-like structure (figure 6(a)), whereas the {110}_pc_-textured CNT ceramic displays ∼45° obliquely aligned block-shaped grains, like a stone-wall structure often seen in Shinto shrines (figure 6(b)). These unique microstructures are the results of *in situ* topochemical formation of a distorted perovskite from layered perovskite templates with the orientation of TiO_6_ octahedrons preserved, and the successive templated growth of the oriented large-sized grains at the expense of small randomly oriented grains formed between reactive templates. A regular perovskite-type structured grains such as relaxer-PbTiO_3_ generally grows with pseudocubic {100} facets [27], for the 〈100〉_pc_ is the slowest growth direction and the {100}_pc_ is the plane with the lowest surface energy and its charge balanced. Similar brick-layer-like microstructure was reported for a {100}_pc_-textured piezoelectric K_0.5_Na_0.5_NbO_3_-based ceramic by Saito *et al* [8]. Templated grains of CNT also grow in an idiomorphic shape with pseudocubic {100} facets, leading to the unique microstructures, as schematically included in figure 6. It should be noted that the horizontal grain size of the {100}_pc_-textured CNT is only a little larger than the diameter of the original CT template while an exaggerated grain growth occurred in the {110}_pc_-textured CNT. The former result suggested that the grain size could be controlled for {100}_pc_-textured perovskite-type ceramics by the size and amount of template particles in the RTGG processing because the templated grains with 〈100〉_pc_ orientation grow with {100}_pc_ facets, meet each other and stop the growth with similar surface energy conditions. We assume an ideal situation: perfect uniaxial alignment and random in-plane orientation of template-originated grains. In a {100}_pc_-textured perovskite, meeting of vertically growing grains with {100}_pc_ facets parallel to each other would form a {100}_pc_ grain boundary for both grains with no further growth. Meeting of horizontally growing grains with {100}_pc_ facets with an arbitrary angle would form a thermodynamically stable grain boundary such as a twin boundary. For a {110}_pc_-textured perovskite, on the other hand, such simple meetings of two {100}_pc_ facets rarely occur, resulting in the significant grain growth at the expense of other template-originated grains.

The NT–F–CNT ceramic exhibited, however, only a weak {110}_pc_ texture. It is attributed to the introduction of pre-synthesized perovskite CaTiO_3_. Regular perovskite phase is thermodynamically more stable than layered perovskite in general and thus diffusion-out of Nd from Nd_2_Ti_2_O_7_ templates for CaTiO_3_ particles was possibly promoted rather than diffusion-in of Ca from CaTiO_3_ into Nd_2_Ti_2_O_7_ templates, which is required for the grain growth of reactive templates.

### 4.3. Microwave dielectric properties of ceramics

**Figure 7.**
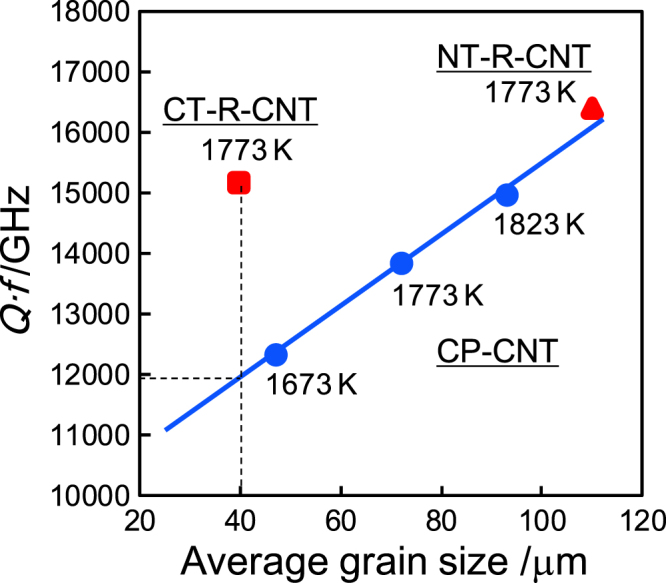
The *Q* · *f* values for textured (red square and triangle) and non-textured (blue circles) CNT ceramics as a function of average grain size.

Table 3 lists *∊*_r_ and *Q* · *f* values for the textured and non-textured CNT ceramics. All the ceramics exhibited nearly identical relative permittivity ranging 99–102, which has a good agreement with the value (∼108) of conventionally processed Ca_0.61_Nd_0.26_TiO_3_ ceramics reported by Liang *et al* [28]. Similar *τ*_f_ values (∼225 ppm K^−1^) regardless of the processing routes assure the similar composition of the three specimens sintered at 1773 K. Origins of a microwave dielectric loss are generally considered as; (1) material itself, (2) crystalline defects, and (3) microstructure of a ceramic including pores and grain boundaries. Microwave dielectric loss in ceramics generally increases as the porosity increases or the grain size decreases. The *Q* · *f* values for the conventionally processed CNT ceramics, CP-CNT, increased as the sintering temperature increased. It is because the ceramics sintered at higher temperature have larger grain size and thus fewer grain boundaries, which resulted in reduced scattering of electric field. Apparent dependence of the *Q* · *f* value on the grain size for the CP-CNT ceramics, as plotted in figure 7, reveals that the grain boundary scattering is the major determining factor for the dielectric loss in this series of CNT ceramics because they have similar relative densities and compositions but different microstructures.

**Table 3.** Dielectric properties of CNT ceramics.

Batch	Sintering temperature (K)	Relative permittivity	*Q* · *f* (GHz)	*τ*_f_, ^a^ (ppm K^−1^)
CP-CNT	1673	99.8	12 340	228
CP-CNT	1773	99.6	13 830	not measured
CP-CNT	1823	99.8	14 970	not measured
CT–R–CNT	1773	101.6	15 140	225
NT–R–CNT	1773	99.8	16 395	222

a Temperature coefficient of resonant frequency was measured between 198 and 253 K.

The {100}_pc_- and {110}_pc_-textured CNT ceramics exhibited ∼10 and ∼20% higher *Q* · *f* values, respectively, than the conventionally processed specimen at the same sintering temperature (1773 K). The high *Q* · *f* value for the {110}_pc_-textured CNT can be attributed to the large grain size as shown in figure 7 although even higher property is expected if the density is identical to the non-textured. It is obvious, however, that the {100}_pc_-textured CNT ceramic exhibited 27% larger figure of merit than non-textured with the same grain size on the extrapolated line. Although the electric field and orientation axis were not in the same direction, three-dimensional randomness were reduced to two-dimensional randomness along the electric field in the textured CNT ceramics. Especially for the {100}_pc_-textured CNT ceramic, grain boundaries are, ideally, either perpendicular or parallel to the cylindrical axis of the ceramic specimen. In other words, electric field mostly penetrates {*hk*0}_pc_-type grain boundary planes which are parallel to the cylindrical axis. Simpler geometrical relationships between grains in the {100}_pc_-textured CNT ceramic could also lead to reduced residual stresses and reduced scattering at grain boundaries along the electric field than in the randomly oriented polycrystals. Compared with the {100}_pc_-texture, {110}_pc_-textured ceramic must have more randomly oriented grain boundary distribution along the electric field.

## 5. Conclusions

This paper exhibits texture design and fabrication of bulk ceramics with two different pseudocubic preferred orientation directions. Texture-dependent microwave dielectric characteristic was also suggested in this study with an improved figure of merit for the {100}_pc_-textured ceramic when compared with randomly oriented. It should be emphasized that the design and fabrication of favorable grain boundary orientation would be a key for microwave dielectric ceramics whereas crystallographic orientation is important for piezoelectric ceramics. Grain boundary orientation is also significantly important for ceramics with electrical conductivity [29]. Advancement in RTGG method would give ceramic engineers more freedom in design and fabrication of high performance devices regardless of the availability of a bulk single crystal with a desired composition. ‘Texture engineering’ for regular perovskite-type materials could enable polycrystalline ceramics with tailored grain and boundary orientations, leading to unique and enhanced properties for various functional devices.
